# Lipid peroxidation and sarcopenia: molecular mechanisms and potential therapeutic approaches

**DOI:** 10.3389/fmed.2025.1525205

**Published:** 2025-02-03

**Authors:** Yifan Lu, Tiao Li, Yang Shu, Chengyin Lu, Zhiqiang Luo, Jingrui Wang, Hui Xiong, Wangyang Li

**Affiliations:** ^1^Department of Orthopedics, The Second Affiliated Hospital of Hunan University of Chinese Medicine, Changsha, China; ^2^Department of Graduate School, Hunan University of Chinese Medicine, Changsha, China; ^3^Department of Respiratory Medicine, Xiangya Hospital of Central South University, Changsha, China; ^4^Department of Orthopedics, The First Affiliated Hospital of Hunan University of Chinese Medicine, Changsha, Hunan, China

**Keywords:** sarcopenia, lipid peroxidation, oxidative stress, antioxidants, therapeutic strategies

## Abstract

Sarcopenia is an age-related condition characterized by the progressive loss of skeletal muscle mass and strength. With the global aging population, its incidence is rapidly increasing. Lipid peroxidation is a critical biochemical process that generates reactive oxygen species (ROS), leading to the destruction of muscle cell structure and function. It plays a pivotal role in the onset and progression of sarcopenia. This review summarizes the mechanisms by which lipid peroxidation contributes to sarcopenia, with a focus on its regulatory effects on cell membrane damage, mitochondrial dysfunction, and cell death. In addition, we discuss the protective role of antioxidant factors such as GPX4 (glutathione peroxidase 4) and antioxidant peptides like SS peptides in mitigating lipid peroxidation and delaying the progression of sarcopenia. Finally, the potential of various strategies, including natural compounds, supplements, natural extracts, and lifestyle interventions, in inhibiting lipid peroxidation and promoting muscle health is explored.

## 1 Introduction

Sarcopenia is a progressive and systemic loss of skeletal muscle mass and strength associated with aging, significantly affecting the quality of life and functional independence of elderly individuals. It is also linked to adverse health outcomes, such as an increased risk of falls, fractures, hospitalization, and mortality (1). With the global aging population, sarcopenia has become an increasingly serious public health issue. Despite its substantial impact, a universally established diagnostic standard is still lacking. Current epidemiological studies indicate a global prevalence of sarcopenia ranging from 10% to 27%, with a prevalence of 8% to 36% in individuals under 60 years of age and 10% to 27% in those aged 60 and above (2). Additionally, elderly women are generally at a higher risk of developing sarcopenia than men (3), possibly due to factors such as decreased bone density (4), hormonal changes, and physiological differences (5). While sarcopenia is common among older adults, it can also occur in middle-aged and even young individuals (6), and is frequently observed in patients with chronic kidney disease, cancer, diabetes, and chronic obstructive pulmonary disease (COPD) (7–10). The prevalence of sarcopenia varies widely, from 18% in diabetic patients to 66% in those with inoperable esophageal cancer (11). These conditions not only accelerate muscle loss but may also exacerbate muscle degeneration through mechanisms such as inflammation, metabolic disorders, and malnutrition (12, 13). Although the etiology of sarcopenia is complex, lipid peroxidation has emerged as a key factor in its onset and progression, attracting growing attention.

Lipid peroxidation involves a series of chemical reactions in which lipid molecules, particularly polyunsaturated fatty acids (PUFAs), are oxidatively attacked by free radicals or non-radical species in the cell membrane or intracellular structures. This process generates lipid radicals and peroxides, which damage the cell membrane structure and function, triggering a chain reaction that further impairs cellular function and induces apoptosis (14). Excessive lipid peroxidation is considered a central mechanism in various diseases. The resulting oxidative stress and cell death or necrosis are particularly prominent in the context of metabolic disorders, inflammation, and aging. Studies have shown that lipid peroxidation is closely associated with the pathogenesis of cardiovascular diseases, neurodegenerative diseases, metabolic disorders, and sarcopenia (15–17).

Lipid peroxidation is a free radical-driven oxidation of fatty acids, leading to damage of cell membranes and organelles. Cells have endogenous defense mechanisms to counteract this oxidative damage. The main defense mechanisms include antioxidant enzyme systems (such as superoxide dismutase, catalase, and glutathione peroxidase) and non-enzymatic antioxidants (such as glutathione, vitamin E, and vitamin C) (18–21). These mechanisms protect cells from oxidative damage by scavenging reactive oxygen species (ROS) and neutralizing lipid peroxidation products. However, under aging or chronic disease conditions, these endogenous defense mechanisms may be impaired, leading to elevated lipid peroxidation levels and exacerbating cellular damage, potentially contributing to diseases such as muscle atrophy.

In sarcopenia, lipid peroxidation may impact muscle health through several pathways. Firstly, lipid peroxidation products directly damage muscle cell membranes, leading to apoptosis and muscle loss (22). Secondly, lipid peroxidation products can induce inflammation and oxidative stress, further exacerbating muscle damage (23). Additionally, lipid peroxidation influences sarcopenia through various mechanisms, including metabolic disorders (24), ferroptosis (25), mitochondrial dysfunction (26), autophagy and apoptosis (27), extracellular matrix remodeling (28), cell signaling pathways (29), as well as lifestyle and nutritional factors (30).

This review summarizes the current research on lipid peroxidation and sarcopenia, including the molecular mechanisms by which lipid peroxidation influences muscle atrophy, protective mechanisms that reduce lipid peroxidation in slowing sarcopenia progression, and lipid peroxidation-based therapeutic strategies for sarcopenia. Finally, we will summarize recent findings and propose future research directions and potential interventions, offering new insights and approaches for the clinical treatment and prevention of sarcopenia.

## 2 Molecular mechanisms by which lipid peroxidation affects muscle atrophy

**Figure 1:**
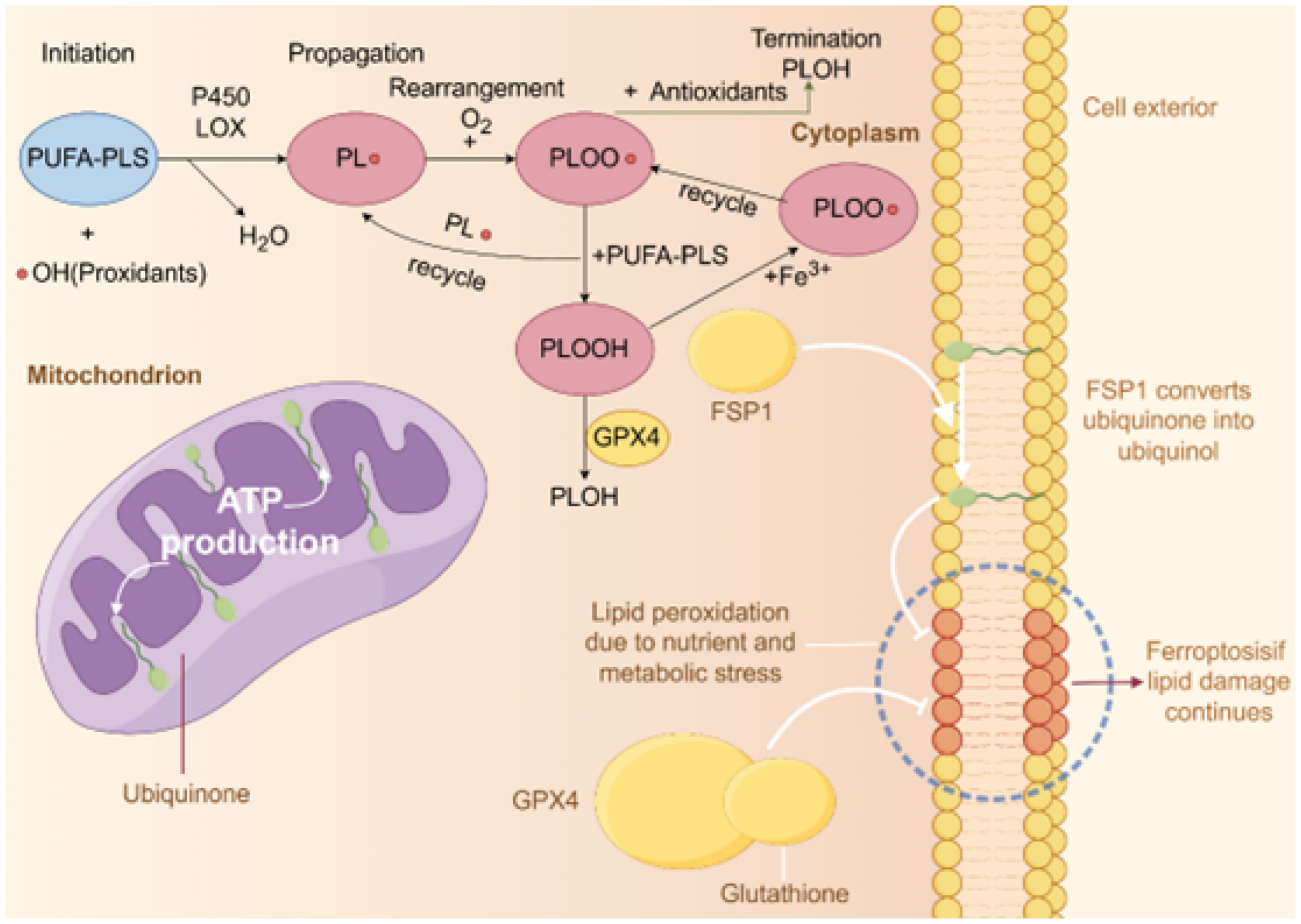
Diagram of lipid peroxidation and antioxidant mechanisms. This figure illustrates the three main stages of intracellular lipid peroxidation and corresponding antioxidant mechanisms: initiation, propagation, and termination. In the initiation stage, polyunsaturated phospholipids (PUFA-PLs) react with hydroxyl radicals (•OH) to form lipid radicals (PL•) under the catalysis of P450 and LOX enzymes. During propagation, PL• reacts with oxygen (O_2_) to produce lipid peroxyl radicals (PLOO•), which further react with other PUFA-PLs to form lipid hydroperoxides (PLOOH), a process that can be promoted by iron ions (Fe^3+^). In the termination stage, antioxidants such as GPX4 and glutathione reduce PLOO• to stable products (PLOH), ending the chain reaction and preventing further lipid peroxidation. Additionally, the figure shows cellular and mitochondrial structures. In mitochondria, ubiquinone is converted to ubiquinol by FSP1, which inhibits lipid damage caused by ferroptosis. Nutritional and metabolic stress can lead to increased lipid peroxidation, and if antioxidant defenses are insufficient, this may trigger ferroptosis.

Free radicals, particularly reactive oxygen species (ROS) and reactive nitrogen species (RNS), are the primary inducers of lipid peroxidation. The mechanism of lipid peroxidation typically occurs in three stages: initiation, propagation, and termination (Figure 1). Initially, free radicals abstract hydrogen atoms from the fatty acid chain, forming lipid radicals and initiating the reaction. This reaction primarily occurs in polyunsaturated fatty acids (PUFAs) due to their multiple double bonds, which make them more susceptible to free radical attack (31). During the propagation stage, the generated lipid radicals react with oxygen molecules to form lipid peroxides. These peroxides further attack other fatty acids, generating new lipid radicals, and continuing the chain reaction (32). This process continues until antioxidants, such as vitamin E or glutathione, intervene to terminate the reaction. In the termination stage, antioxidants stabilize lipid radicals by donating hydrogen atoms, thereby preventing further lipid peroxidation (33).

### 2.1 Membrane damage induced by lipid peroxidation

Lipid peroxidation is one of the key mechanisms in muscle atrophy. The cell membrane plays a critical role in muscle cell function, particularly in muscle contraction, signal transduction, and metabolism. Lipid peroxidation not only damages the structure and function of the cell membrane but also triggers a series of secondary pathological changes, including cell death and muscle atrophy. The lipid peroxidation process mainly involves the oxidation of fatty acid double bonds, resulting in the formation of hydrogen peroxide, lipid peroxides, and their derivatives (e.g., α,β-unsaturated aldehydes). These products chemically damage membrane lipids, membrane proteins, and cholesterol, especially since membrane phospholipids are rich in unsaturated fatty acids, making them more vulnerable to free radical attack. This attack alters the membrane's conformation and fluidity, compromising its integrity (34, 35). These changes increase membrane permeability, affect membrane protein function, and lead to ion imbalance, particularly the influx of calcium ions, which in turn causes muscle fiber damage (36).

Membrane proteins, such as the sodium-potassium pump (Na+/K+ ATPase) and calcium ATPase (SERCA), are crucial molecules in maintaining the cell's electrochemical gradient and material transport. Lipid peroxidation alters the structure and function of these proteins, resulting in elevated intracellular calcium concentrations, promoting uncoordinated muscle contraction, and leading to cellular damage, thus exacerbating muscle atrophy (36). Additionally, lipid peroxidation products, such as 4-HNE and MDA, have immune-activating properties and can induce the recruitment and activation of immune cells, such as macrophages. The inflammatory factors (e.g., TNF-α, IL-6) released by these immune cells further aggravate membrane damage and cellular destruction, creating a vicious cycle that accelerates the progression of sarcopenia (37, 38). The impact of lipid peroxidation on membrane damage is not limited to the cell membrane; mitochondrial membranes may also be affected. Therefore, membrane damage induced by lipid peroxidation is one of the key pathological mechanisms driving sarcopenia. Inhibiting lipid peroxidation or clearing its products may serve as an effective strategy for treating muscle atrophy.

### 2.2 The relationship between mitochondrial dysfunction and lipid peroxidation

Mitochondrial-associated lipid peroxidation plays a crucial role in the progression of sarcopenia. Mitochondria, as the powerhouses of the cell, are involved in ATP synthesis, metabolic regulation, and cell survival. Lipid peroxidation damages mitochondrial membranes and disrupts mitochondrial function, leading to insufficient energy supply in muscle cells, which exacerbates muscle atrophy and the onset of sarcopenia (39). The outer and inner mitochondrial membranes are rich in unsaturated fatty acids, and lipid peroxidation products, such as 4-hydroxy-2-nonenal (4-HNE) and malondialdehyde (MDA), alter the fluidity and permeability of the membrane by binding with membrane lipids. This, in turn, induces mitochondrial permeability transition (MPT). MPT leads to intracellular calcium ion accumulation, triggering mitochondrial dysfunction. Lipid peroxidation also directly affects ATP synthase and membrane proteins, inhibiting ATP synthesis, disrupting energy metabolism, and further accelerating the decline in muscle cell function (40, 41).

Mitochondria are major sources of free radicals, and lipid peroxidation exacerbates oxidative stress by increasing the generation of reactive oxygen species (ROS), damaging mitochondrial membranes and DNA, and weakening repair capabilities. Excessive ROS accumulation not only causes membrane damage but also induces mitochondrial DNA damage, alters gene expression, and promotes muscle atrophy. Lipid peroxidation products can also activate intrinsic apoptotic pathways within mitochondria, leading to cell death and further exacerbating sarcopenia (42, 43).

Lipid peroxidation can also inhibit mitochondrial autophagy. Damaged mitochondria are not removed in time and accumulate to form “waste” mitochondria, which release more ROS, accelerating muscle atrophy (7). Moreover, studies show that increased mitochondrial ROS (mtROS) can damage the neuromuscular junction (NMJ), leading to contraction dysfunction. In mSod2KO mice, despite an increase in muscle mass, significant reductions in contraction force were observed due to elevated mitochondrial ROS and lipid peroxidation, highlighting a new role of mitochondrial oxidative stress in maintaining muscle mass via fiber branching (44). Therefore, lipid peroxidation may disrupt mitochondrial membranes, induces oxidative stress, activates apoptotic pathways, and inhibits autophagy, leading to mitochondrial dysfunction and exacerbating muscle atrophy. Improving mitochondrial function or activating mitochondrial autophagy may serve as potential therapeutic strategies to alleviate sarcopenia.

### 2.3 Lipid peroxidation-induced muscle cell death and atrophy

Lipid peroxidation is a byproduct of cellular responses to oxidative stress. In addition to damaging muscle cell membranes and mitochondrial function, lipid peroxidation exacerbates muscle atrophy by inducing muscle cell death. Muscle cell death mainly occurs through apoptosis, necrosis, and autophagy, resulting in muscle fiber loss and promoting the progression of sarcopenia.

Apoptosis is a programmed cell death process. Lipid peroxidation increases ROS generation and damages cell membranes, activating a series of pro-apoptotic signaling molecules (e.g., caspase-3, p53), which lead to muscle cell apoptosis. Lipid peroxidation products, such as 4-HNE and MDA, activate the p53 pathway, inhibit Bcl-2 (an anti-apoptotic protein), activate Bax (a pro-apoptotic protein), increase mitochondrial membrane permeability, release cytochrome c, and initiate the caspase cascade, ultimately leading to cell death (45–47).

Moreover, lipid peroxidation is closely associated with necrotic cell death. Necrosis is an acute form of cell death caused by membrane damage, which is accompanied by cell swelling, membrane rupture, and leakage of cellular contents. Lipid peroxidation induces membrane damage, leading to uncontrolled calcium ion influx and exacerbating intracellular calcium accumulation, thereby triggering necrosis. Necrotic cells release danger signals (such as high-mobility group box-1 (HMGB1), ATP, etc.), which activate inflammatory responses and aggravate oxidative stress and muscle damage (48–50).

Autophagy is a self-protective mechanism of cells, but lipid peroxidation disrupts the function of autophagy-related genes (e.g., Beclin-1, LC3), leading to autophagic decline, preventing the clearance of damaged mitochondria and protein complexes, and accelerating muscle cell death (51–53).

Lipid peroxidation also exacerbates muscle atrophy by activating muscle protein degradation pathways (e.g., the ubiquitin-proteasome pathway, calcium-dependent pathways). Elevated ROS stimulate protein degradation pathways, resulting in reduced protein synthesis and increased degradation, thus accelerating muscle fiber loss. Furthermore, lipid peroxidation products promote inflammation through cytokines (e.g., IL-6, TNF-α), further exacerbating metabolic dysregulation and muscle atrophy (54, 55).

Additionally, lipid peroxidation products play distinct roles under specific pathological conditions. For example, cholesterol oxidation derivatives, such as 7β-hydroxycholesterol and 7-ketocholesterol, are significantly elevated in sarcopenia patients and exert cytotoxic effects on myoblasts and myotubes (56). These oxidized sterols exacerbate muscle atrophy by inducing inflammation and cell death. Antioxidants, such as α-tocopherol and pistachio oil, effectively reduce oxidized sterol-induced cytotoxicity and decrease the release of inflammatory factors, suggesting that antioxidant therapy may help slow the progression of sarcopenia. In conclusion, lipid peroxidation-induced muscle cell death and atrophy are key factors in the development of sarcopenia. Regulating lipid peroxidation may become an important strategy for the future treatment of sarcopenia.

### 2.4 Factors that aggravate lipid peroxidation

Lipid peroxidation drives the pathological progression of sarcopenia through free radical reactions in conditions such as aging, disuse atrophy, denervation, and chronic diseases. The accumulation of lipid peroxidation products is closely associated with multiple factors, including genetic variation, denervation, immobilization and remobilization, chronic diseases, nutritional deficiencies, iron overload, alcoholic myopathy, and others. This section explores these aggravating factors, their effects in specific pathological environments, and potential intervention strategies (Table 1).

**Table 1:** Lipid peroxidation and its impact on muscle atrophy under various conditions.

**Factor**	**Mechanism/effect**	**Relevant findings**	**References**
Genetic variation	ALDH2 gene mutation (rs671, ALDH2^*^2) leads to 4-HNE accumulation in muscles, disrupting metabolism and causing muscle atrophy.	Vitamin E supplementation reduces 4-HNE levels and alleviates muscle atrophy, indicating lipid peroxidation's role in genetically susceptible sarcopenia.	(57)
Denervation	Denervation induces increased cPLA2 activity, leading to high lipid peroxidation products (LOOH), exacerbating muscle atrophy.	Inhibition of cPLA2 reduces LOOH production, mitigates atrophy, and maintains muscle fiber size.	(58, 59)
Immobilization and remobilization	Immobilization increases lipid peroxidation in muscles and blood, peaking in initial and remobilization stages.	Elevated lipid peroxidation and reduced glutathione levels during immobilization and remobilization exacerbate muscle damage and atrophy.	(60)
Chronic diseases (e.g., heart failure)	Increased lipid peroxidation products (4-HNE, acrolein) in heart failure models exacerbate muscle atrophy.	Decreased detoxification of toxic aldehydes worsens muscle atrophy in heart failure models.	(61)
Nutritional deficiencies	Vitamin D deficiency exacerbates sarcopenia by increasing lipid peroxidation. Protein malnutrition also increases lipid peroxidation.	Smoking and vitamin D deficiency elevate lipid peroxidation, accelerating muscle loss and atrophy. In liver cirrhosis, low-protein diet worsens fiber atrophy, associated with increased iron content in muscles.	(62, 63)
Moderate iron load	Moderate iron load induces lipid peroxidation and impairs skeletal muscle function.	Moderate iron load increases non-heme iron in muscles, impairing contractile function despite unchanged muscle mass.	(64)
Alcoholic myopathy	Alcohol and protein malnutrition increase lipid peroxidation, leading to muscle fiber atrophy.	The synergistic effect of alcohol and protein deficiency significantly elevates lipid peroxidation, exacerbating muscle atrophy, especially with iron overload.	(65)
Oxidative stress and muscle damage	Increased oxidative stress leads to higher lipid peroxidation, weakening antioxidant defenses and aggravating muscle atrophy.	In hindlimb unloading models, oxidative stress impairs antioxidant capacity, increases lipid peroxidation, and worsens muscle damage. Dysregulation of heat shock proteins further exacerbates muscle atrophy.	(66, 67)
Drug effects (e.g., dexmedetomidine)	Some drugs like dexmedetomidine can induce lipid peroxidation, leading to muscle dysfunction.	Dexmedetomidine increases lipid peroxidation in the diaphragm but does not alleviate ventilator-induced diaphragm dysfunction, highlighting the central role of oxidative stress.	(68)
Sensitivity of muscle fiber types to oxidative stress	Different muscle fiber types exhibit varying sensitivities to oxidative stress.	Fast-twitch fibers are more susceptible to lipid peroxidation-induced damage and atrophy compared to slow-twitch fibers, which have higher antioxidant capacity.	(69)

Genetic variation can significantly influence the accumulation of lipid peroxidation products. For example, the ALDH2 gene mutation (rs671, ALDH2^*^2) leads to the accumulation of 4-hydroxy-2-nonenal (4-HNE) in muscles, disrupting metabolic balance and causing muscle atrophy. Supplementation with the antioxidant vitamin E significantly lowers 4-HNE levels and alleviates muscle atrophy, highlighting the role of lipid peroxidation in genetically susceptible sarcopenia (57).

Denervation is another major cause of lipid peroxidation accumulation. In denervation-induced muscle atrophy models, increased activity of cytosolic phospholipase A2 (cPLA2) leads to high levels of lipid peroxidation products, such as lipid hydroperoxides (LOOH), aggravating muscle atrophy. Inhibition of cPLA2 reduces LOOH production, mitigates atrophy, and maintains muscle fiber size. This finding has been validated in several age-related muscle atrophy models (58, 59).

Both immobilization and subsequent remobilization are closely associated with changes in lipid peroxidation. During immobilization, lipid peroxidation in both muscles and blood increases significantly, peaking during the initial stage of immobilization and again during remobilization. The elevated lipid peroxidation during immobilization, coupled with reduced glutathione levels, indicates persistent oxidative stress, which further exacerbates muscle damage and atrophy (60).

Chronic Diseases and Lipid Peroxidation Chronic diseases, such as heart failure, are also associated with the accumulation of lipid peroxidation products. In heart failure mouse models, lipid peroxidation products like 4-HNE and acrolein significantly increase, while pathways for detoxifying these toxic aldehydes decrease, leading to further accumulation and exacerbation of muscle atrophy (61).

Nutritional Deficiencies and Lipid Peroxidation Nutritional deficiencies, particularly vitamin D deficiency, exacerbate sarcopenia by increasing lipid peroxidation and negatively impacting skeletal muscle function. In smoking mouse models, vitamin D deficiency initially does not affect muscle mass but, over time, leads to elevated lipid peroxidation, which, in combination with smoking, accelerates muscle loss and atrophy (62). Furthermore, protein malnutrition is closely linked to increased lipid peroxidation. In a rat model of liver cirrhosis induced by carbon tetrachloride, a low-protein diet exacerbates type IIa fiber atrophy, which is associated with increased iron content in muscles (63).

Moderate iron load can induce lipid peroxidation and impair skeletal muscle function. Research shows that moderate iron load significantly increases non-heme iron levels in skeletal muscles. Although body weight and muscle mass remain unchanged, specific force (tension) decreases, indicating impaired contractile function. This damage is likely caused by oxidative stress, increased protein degradation, and abnormal calcium release mechanisms within muscles (64).

Alcoholic Myopathy and Lipid Peroxidation Alcoholic myopathy is the result of both lipid peroxidation and protein malnutrition, leading to type II muscle fiber atrophy. The synergistic effect of alcohol and protein deficiency significantly increases lipid peroxidation products like malondialdehyde (MDA), which aggravates muscle atrophy. Notably, in animals with protein deficiency, the effects of iron overload and lipid peroxidation are more pronounced, suggesting that oxidative stress and iron overload are key contributors to sarcopenia in alcoholic myopathy (65).

Oxidative Stress and Muscle Damage In a hindlimb unloading model, increased oxidative stress significantly impairs the antioxidant capacity of skeletal muscles, elevating lipid peroxidation levels and making muscles more susceptible to damage and atrophy during prolonged unloading and reloading. This increase in oxidative stress, combined with weakened antioxidant defenses, exacerbates muscle damage (66). Another study confirmed this finding, showing that during hindlimb unloading and reloading, lipid peroxidation in skeletal muscles significantly increases, accompanied by dysregulation of heat shock proteins (HSPs), especially a decrease in Hsp70 and Hsp25 expression, which further aggravates muscle damage and atrophy (67).

Some drugs, such as dexmedetomidine, may have adverse effects on muscles through lipid peroxidation. Dexmedetomidine increases lipid peroxidation levels in the diaphragm of mechanically ventilated rats. Although it has some antioxidant potential, it does not alleviate ventilator-induced diaphragm dysfunction, indicating that oxidative stress and activation of proteolytic pathways play a central role in this process (68).

Sensitivity of Muscle Fiber Types to Oxidative Stress Different muscle fiber types exhibit varying sensitivities to oxidative stress. In a streptozotocin-induced diabetic rat model, lipid peroxidation levels in fast-twitch fibers (such as the extensor digitorum longus) were significantly increased and closely associated with atrophy. In contrast, slow-twitch fibers, like those in the soleus muscle, are more adaptable to oxidative stress and have higher antioxidant enzyme activity, although both fiber types ultimately undergo atrophy. This suggests that fast-twitch fibers are more susceptible to damage induced by oxidative stress (69).

## 3 Protective mechanisms against sarcopenia progression by reducing lipid peroxidation

In the mechanism by which lipid peroxidation contributes to muscle atrophy and functional loss, various protective mechanisms play a crucial role. By reducing lipid peroxidation, maintaining cellular redox balance, or modulating specific signaling pathways, these mechanisms can effectively slow the progression of sarcopenia. Table 2 summarizes these important protective mechanisms, including GPx4, ω-3 polyunsaturated fatty acids, and LPCAT3, which mitigate the negative effects of lipid peroxidation through different pathways. In the following sections, we will discuss each mechanism in detail, along with its potential clinical applications.

**Table 2:** Molecular mechanisms and interventions targeting lipid peroxidation in sarcopenia.

**Molecule/factor**	**Mechanism/effect**	**Key findings/research**	**Reference**
GPX4 (Glutathione Peroxidase 4)	Direct scavenger of lipid peroxides; maintains cellular redox balance and prevents lipid peroxidation chain reactions.	GPX4 reduces lipid peroxides, preventing muscle atrophy. Its expression/activity decreases with aging, contributing to muscle degeneration. Enhancing GPX4 activity alleviates sarcopenia.	(70–74)
ω-3 Polyunsaturated Fatty Acids (n-3 PUFAs)	Anti-inflammatory and antioxidant effects, reduces lipid peroxidation.	Elevated n-3 PUFA levels reduce lipid peroxidation and inflammation, improving muscle function and alleviating cachexia in hypoxic-ischemic injury models.	(75)
LPCAT3 (Lysophosphatidylcholine Acyltransferase 3)	Modulates the Lands cycle, reducing lipid peroxidation in skeletal muscle.	LPCAT3 inhibition reduces lipid hydroperoxides and alleviates muscle atrophy, suggesting a potential target for sarcopenia therapy.	(76)
Kynurenine (KYN)	Increases ROS levels, decreases protein synthesis, and promotes lipid peroxidation.	KYN activation of AhR increases oxidative stress and lipid peroxidation, contributing to sarcopenia. Inhibition of KYN improves muscle fiber size and strength in aged mice.	(77)
SERCA (Sarcoplasmic/Endoplasmic Reticulum Calcium ATPase) Activity	Regulates calcium homeostasis; reduced activity exacerbates muscle atrophy.	Asthma patients exhibit decreased SERCA activity and increased oxidative stress. Enhancing SERCA activity may improve sarcopenia in asthma-associated muscle weakness.	(78)
Iron homeostasis	Iron accumulation increases lipid peroxidation and impairs muscle regeneration.	In aged mice, iron accumulation leads to increased lipid peroxidation. While deferiprone reduces iron levels, it does not significantly improve muscle regeneration.	(79)
Mitochondrial function	Mitochondrial dysfunction and oxidative stress contribute to muscle atrophy.	Overexpression of PGC-1α improves mitochondrial function and alleviates muscle atrophy, offering a potential strategy for age-related sarcopenia.	(80)
Mitochondrial T3 receptor (p43)	Overexpression of p43 induces oxidative stress and lipid peroxidation, contributing to muscle atrophy.	p43 overexpression in mice induces mitochondrial dysfunction, oxidative stress, and muscle atrophy through the ubiquitin-proteasome pathway.	(82)
Disuse atrophy and mitochondrial dysfunction	Reduced mitochondrial respiration and increased oxidative stress during disuse atrophy.	Hindlimb unloading-induced muscle atrophy is associated with impaired mitochondrial function and increased oxidative stress. Maintaining mitochondrial function is critical for muscle recovery.	(83)
Caspase-3	Apoptotic factor activated during muscle atrophy; involved in lipid peroxidation and muscle cell death.	Caspase-3 activation during hindlimb unloading-induced atrophy contributes to muscle damage. Modulation of apoptosis may prevent disuse muscle atrophy.	(84)

### 3.1 GPx4

GPX4 (glutathione peroxidase 4) is an essential member of the antioxidant enzyme family, playing a key role in preventing cellular damage caused by lipid peroxidation. Lipid peroxidation leads to damage to cell membranes and mitochondria, which in turn results in cellular dysfunction and muscle atrophy. As a direct scavenger of lipid peroxides, GPX4 effectively removes harmful lipid peroxides and prevents the propagation of lipid peroxidation chain reactions through reduction processes, thereby maintaining cellular redox balance (70). The protective role of GPX4 becomes particularly important during aging and muscle atrophy. As aging progresses, the accumulation of lipid peroxides increases, causing instability in cell membranes, particularly in muscle cells. During this process, GPX4 expression or activity often decreases, leading to reduced cellular responses to oxidative damage and accelerating muscle degeneration. Studies have shown that enhancing GPX4 activity or overexpression can effectively reduce muscle damage caused by lipid peroxidation and delay the progression of muscle atrophy (71).

Research indicates that GPX4 plays a critical regulatory role in reducing the accumulation of lipid hydroperoxides during denervation-induced oxidative stress. In CuZn superoxide dismutase gene knockout mice, overexpression of GPX4 significantly improves mitochondrial respiratory function, restores contraction strength and membrane excitability in fast-twitch muscle fibers. Additionally, GPX4 enhances the activity of muscle/ER Ca^2+^-ATPase (SERCA), maintaining calcium homeostasis and excitation-contraction coupling, thereby alleviating sarcopenia progression (72). Apart from reducing lipid hydroperoxide levels, GPX4 also regulates the formation of oxidized phospholipids. By decreasing age-related lipid peroxidation and oxidative damage, overexpression of GPX4 offers a promising preventive and therapeutic approach for age-related sarcopenia by maintaining cell membrane integrity and reducing oxidative stress (71).

In a hindlimb suspension model of muscle atrophy, oxidative stress increases, and antioxidant enzyme imbalance is a key factor. Studies have shown that regulating oxidative stress and antioxidant enzyme expression, such as enhancing manganese superoxide dismutase (MnSOD) and GPx activity, can effectively slow the progression of muscle atrophy, providing a new approach to sarcopenia prevention (73).

The accumulation of lipid peroxides is the foundation for lipid peroxides' role in sarcopenia. As aging and disuse atrophy progress, lipid hydroperoxides (LOOH) in muscle tissue significantly increase, promoting muscle atrophy through carbonyl stress. Research indicates that the accumulation of LOOH is associated with insufficient activity of key antioxidant enzyme GPX4. When GPX4 activity decreases, LOOH levels rise, initiating lysosome-dependent and proteasome-independent pathways of atrophy (74). Genetic and pharmacological interventions that neutralize LOOH and its reaction products can effectively prevent muscle atrophy and weakness induced by aging and disuse.

### 3.2 ω-3 polyunsaturated fatty acids

In addition to GPX4, ω-3 polyunsaturated fatty acids (n-3 PUFAs) possess significant anti-inflammatory and antioxidant effects, particularly in mitigating cachexia induced by hypoxic-ischemic brain injury. Transgenic mice with elevated levels of n-3 PUFAs show reduced inflammatory responses and lipid peroxidation after injury, along with improved hypothalamic-pituitary-adrenal (HPA) axis function, suggesting that n-3 PUFAs play a protective role in alleviating metabolic imbalances associated with cachexia (75).

### 3.3 Lysophosphatidylcholine acyltransferase 3

Moreover, inhibiting the Lands cycle by modulating the activity of lysophosphatidylcholine acyltransferase 3 (LPCAT3) in skeletal muscle effectively reduces lipid peroxidation and alleviates muscle atrophy and weakness. In skeletal muscle-specific LPCAT3 knockout mice, lipid hydroperoxide levels were significantly reduced, indicating that suppressing the Lands cycle may be an effective strategy for alleviating sarcopenia (76).

### 3.4 Kynurenine

Kynurenine (KYN), a tryptophan metabolite that increases with age, activates the aryl hydrocarbon receptor (AhR) and increases ROS levels, which are associated with decreased protein synthesis and increased lipid peroxidation. Inhibition of KYN production improves muscle fiber size and strength in aged mice, suggesting that KYN is a key mediator of sarcopenia. Interestingly, KYN's effects on oxidative stress and lipid peroxidation occur independently of direct activation of AhR, making KYN metabolic inhibition a potential preventive approach for sarcopenia (77).

### 3.5 SERCA activity

Sarcopenia also occurs in patients with asthma, closely linked to reduced SERCA (sarcoplasmic/endoplasmic reticulum calcium ATPase) activity. Asthma patients exhibit decreased SERCA activity, increased oxidative stress, and lipid peroxidation, leading to muscle weakness and atrophy. Enhancing SERCA activity may provide a potential strategy for improving sarcopenia associated with asthma (78).

### 3.6 Iron homeostasis regulation

Iron accumulation in aged skeletal muscle is another factor that leads to increased lipid peroxidation and impaired regenerative capacity. In aged mice after ischemia-reperfusion (IR) injury, iron levels rise significantly and are associated with lipid peroxidation. While deferiprone (DFP) treatment can reduce iron levels in muscle, it does not significantly improve muscle regeneration, underscoring the importance of iron homeostasis regulation for muscle health and regeneration (79).

### 3.7 Mitochondrial function

Mitochondrial dysfunction plays a crucial role in the occurrence and progression of sarcopenia. Increased expression of mitochondrial autophagy-related proteins is closely associated with age-related mitochondrial dysfunction. However, overexpression of PGC-1α reduces autophagy marker levels, improves mitochondrial metabolic function and antioxidant capacity, thereby alleviating muscle atrophy, offering a potential approach to age-related sarcopenia (80). During cancer cachexia, mitochondrial dysfunction also plays a role in muscle atrophy. Although no significant lipid peroxidation is observed in cancer-associated muscle atrophy, increased mitochondrial protein carbonylation impairs mitochondrial function, suggesting that regulating mitochondrial phospholipid biosynthesis pathways may be an effective therapeutic target for cancer-associated muscle wasting (81).

Overexpression of the mitochondrial T3 receptor (p43) has also been shown to induce lipid peroxidation and lead to muscle atrophy. In p43-overexpressing mice, mitochondrial mass initially increases, but mitochondrial DNA content declines over time, impairing mitochondrial function. This overexpression triggers significant oxidative stress, increasing lipid and protein oxidation levels, and ultimately exacerbates atrophy through the ubiquitin-proteasome pathway, likely stimulated by muscle-specific E3 ligases Atrogin-1/MAFbx and MuRF1. This suggests that dysregulation of the direct T3 mitochondrial pathway may be an important driver of sarcopenia, emphasizing the need for precise control of p43 expression (82).

Moreover, mitochondrial dysfunction significantly affects muscle function recovery after disuse atrophy. In the hindlimb unloading-induced atrophy model, reduced mitochondrial respiration, calcium retention capacity, and increased oxidative stress are closely associated with muscle function decline, suggesting that maintaining mitochondrial function is critical for muscle recovery (83).

### 3.8 Caspase-3

Under both normal and low-temperature conditions, apoptotic factors such as caspase-3 are activated during hindlimb unloading-induced atrophy, accompanied by DNA fragmentation and lipid peroxidation. Modulating the apoptosis pathway may play an important role in preventing disuse muscle atrophy (84).

## 4 Targeted therapeutic strategies for sarcopenia based on lipid peroxidation

In the treatment strategies for muscle atrophy related to lipid peroxidation, in addition to natural compounds, various interventions such as supplements, natural extracts, and lifestyle modifications are also included. These strategies work through different mechanisms to slow down or reverse muscle damage caused by lipid peroxidation. Table 3 summarizes these therapeutic strategies, including natural compounds, supplements, natural extracts, and other intervention methods, along with their mechanisms of action. Next, we will discuss each of these strategies in detail, exploring their specific effects, mechanisms, and potential clinical applications.

**Table 3:** Natural compounds and interventions targeting oxidative stress and muscle atrophy.

**Intervention type**	**Compound/agent**	**Mechanism/effect**	**Key findings/research**	**Reference**
Natural compounds	Astaxanthin (AX)	Antioxidant, reduces mitochondrial ROS, inhibits mitochondrial apoptosis, prevents muscle atrophy.	AX prevents muscle atrophy by reducing ROS production and enhancing mitochondrial function.	(85)
	Fucoxanthin (Fx)	Reduces muscle mass loss, inhibits lipid peroxidation, enhances mitochondrial function.	Fx alleviates glucocorticoid-induced muscle atrophy by reducing oxidative stress and improving mitochondrial function.	(86)
	Quercetin	Reduces ubiquitin ligases, lipid peroxidation, alleviates muscle atrophy.	Quercetin preserves muscle mass and function by reducing oxidative stress in disuse models.	(87)
	Resveratrol	Reduces lipid peroxidation, enhances antioxidant enzyme activity, improves redox balance.	Resveratrol prevents muscle strength loss and alleviates oxidative stress in disuse models.	(88)
	S-allyl cysteine (SAC)	Reduces ROS levels, inhibits pro-inflammatory cytokines, alleviates muscle atrophy.	SAC alleviates muscle atrophy by modulating oxidative stress and reducing inflammation.	(89)
	Camphene	Reduces ROS levels, improves muscle atrophy in starvation models.	Camphene reduces oxidative stress induced by starvation and hydrogen peroxide, improving muscle function in animal models.	(90)
	Geranylgeraniol (GGOH) + Green Tea Polyphenols (GTP)	Modulates gut microbiota, reduces fat accumulation, increases skeletal muscle mass.	The combination reduces muscle mass loss and enhances muscle function in high-fat diet models.	(91)
Supplements	Glutamine (GLN)	Increases heat shock protein expression, reduces protein degradation, balances redox status.	GLN supplementation improves muscle protein turnover and reduces atrophy in rat models.	(92)
	Pyrroloquinoline Quinone (PQQ)	Reduces lipid peroxidation, alleviates muscle atrophy and pain in nerve injury models.	PQQ reduces oxidative stress and alleviates muscle atrophy and pain in chronic injury models.	(93)
	Vitamin E	Reduces lipid peroxidation and protein carbonylation, protects against glucocorticoid-induced muscle atrophy.	Vitamin E supplementation mitigates muscle atrophy and oxidative damage in glucocorticoid-treated animals.	(94)
Natural extracts	Handelin	Increases IGF-1 expression, inhibits NF-κB activation, reduces lipid peroxidation.	Handelin protects against cachexia and age-related muscle atrophy by improving muscle function and reducing inflammation.	(95)
	Schisandra Extract	Modulates protein synthesis genes, inhibits lipid peroxidation, enhances antioxidant enzyme activity.	Schisandra extract reduces oxidative stress and enhances muscle adaptation post-exercise by modulating antioxidant pathways.	(96)
	Eriocitrin (Lemon Peel Extract)	Reduces lipid peroxidation, alleviates disuse muscle atrophy.	Eriocitrin reduces muscle atrophy by inhibiting key atrophy-related proteins like Atrogin-1 and MuRF-1.	(97)
	Chlorella	Restores muscle mass, alleviates mitochondrial defects.	Long-term Chlorella intake restores muscle mass in mitochondrial defect models, showing potential for treating age-related sarcopenia.	(98)
Lifestyle interventions	Exercise Training	Reduces lipid hydroperoxides, inhibits UPS activation, reduces oxidative stress, prevents atrophy.	Exercise training improves muscle function and prevents oxidative damage in heart failure patients and other atrophy models.	(99)
	Electrical Stimulation	Reduces lipid peroxidation, restores antioxidant enzyme activity, prevents denervation-induced atrophy.	Electrical stimulation reduces oxidative stress and muscle atrophy caused by denervation.	(100)
Other	Liproxstatin-1	Scavenges lipid hydroperoxides, prevents muscle atrophy and mitochondrial damage.	Liproxstatin-1 alleviates muscle atrophy by reducing lipid peroxidation and mitochondrial damage in denervation models.	(101)
	Neurobolil and thyroxin	Stimulates tissue growth, enhances antioxidant activity, reduces muscle atrophy.	Neurobolil and thyroxin reduce muscle atrophy by enhancing antioxidant defenses and stimulating muscle regeneration.	(102)
	He-Ne Laser Irradiation	Increases NO levels, reduces lipid peroxidation, slows DMD progression.	He-Ne laser irradiation alleviates oxidative stress and lipid peroxidation in DMD models, potentially slowing disease progression.	(103)
	SS Peptides (Sulfated Saccharide Peptides)	Inhibits oxidative damage, enhances antioxidant enzyme systems like GPX4, reduces lipid peroxidation.	SS peptides reduce oxidative stress and muscle atrophy by modulating antioxidant defenses and improving cellular health, showing promise in sarcopenia treatment.	(104, 105)

### 4.1 Natural compounds

Among various interventions targeting oxidative stress and muscle atrophy, natural compounds play a central role. For instance, astaxanthin (AX), a carotenoid with strong antioxidant properties, can reduce mitochondrial ROS production, enhance mitochondrial respiration, inhibit mitochondrial-mediated apoptosis, and effectively prevent muscle atrophy (85).

Fucoxanthin (Fx) also reduces muscle mass loss and oxidative stress induced by glucocorticoids in a dexamethasone-induced muscle atrophy model, highlighting its potential in preventing glucocorticoid-induced muscle atrophy by inhibiting lipid peroxidation and enhancing mitochondrial function (86).

Quercetin and resveratrol have shown potential in combating disuse muscle atrophy. Quercetin reduces the expression of ubiquitin ligases and lipid peroxidation, effectively alleviating muscle atrophy in disuse suspension models while preserving muscle mass and function (87). Resveratrol significantly reduces lipid peroxidation levels, alleviates oxidative stress, and prevents muscle strength loss by enhancing antioxidant enzyme activity in disuse models, showing promise for improving redox balance (88).

Similarly, S-allyl cysteine (SAC), an organic sulfur compound derived from garlic, demonstrates broad antioxidant and immunomodulatory effects by lowering ROS levels and inhibiting pro-inflammatory cytokines (e.g., IL-6 and myostatin), thus alleviating muscle atrophy (89).

Camphene also reduces ROS levels induced by starvation and hydrogen peroxide in both *in vitro* and *in vivo* models, improving muscle atrophy induced by starvation in rats. Its antioxidant activity offers significant protective effects on skeletal muscle atrophy (90). Moreover, the combined use of geranylgeraniol (GGOH) and green tea polyphenols (GTP) reduces fat accumulation, increases skeletal muscle mass, and alleviates the adverse effects of high-fat diets on skeletal muscles through modulation of the gut microbiota (91).

### 4.2 Supplements

In rat models, oral supplementation of glutamine (GLN) increases the expression of heat shock protein 70 (HSP70), balances intracellular redox status, and reduces skeletal muscle protein degradation and atrophy (92).

Pyrroloquinoline quinone (PQQ), a natural redox cofactor, shows analgesic effects in chronic constriction injury (CCI) models. PQQ reduces pro-inflammatory mediators and lipid peroxidation products, thus alleviating muscle atrophy and pain associated with nerve damage (93).

Vitamin E, another potent antioxidant, protects against glucocorticoid-induced muscle atrophy (e.g., dexamethasone) by reducing lipid peroxidation and protein carbonylation, thereby reducing muscle atrophy (94).

### 4.3 Natural extracts

Other natural compounds, such as handelin and Schisandra extracts, show significant anti-atrophy effects. Handelin, derived from chrysanthemums, protects against cachexia and age-related muscle atrophy by increasing the expression of insulin-like growth factor (IGF-1) and inhibiting the activation of NF-κB. These effects help preserve muscle mass, improve muscle function, and maintain structural integrity by regulating inflammatory factors and reducing lipid peroxidation (95). Schisandra extract modulates genes involved in protein synthesis and degradation, inhibits lipid peroxidation, enhances endogenous antioxidant enzyme activity, reduces oxidative stress-related muscle damage, and enhances post-exercise muscle adaptation (96).

A compound in lemon peel, Eriocitrin, reduces lipid peroxidation and alleviates disuse muscle atrophy in denervated mice by inhibiting the expression of the ubiquitin ligases Atrogin-1 and MuRF-1 (97).

Long-term intake of Chlorella can restore muscle mass in mitochondrial aldehyde dehydrogenase 2-deficient mice and alleviate skeletal muscle atrophy associated with mitochondrial defects (98).

### 4.4 Lifestyle interventions

Exercise training is an effective strategy against muscle atrophy, particularly in heart failure (HF) patients. Exercise training improves skeletal muscle function by restoring lipid hydroperoxide and protein carbonyl levels, inhibiting excessive activation of the ubiquitin-proteasome system (UPS), reducing oxidative stress, and preventing skeletal muscle atrophy (99).

*In vivo* electrical stimulation significantly reduces lipid peroxidation levels following denervation, restores antioxidant enzyme activity, and effectively prevents oxidative damage and muscle atrophy caused by denervation (100).

### 4.5 Other

In denervation-induced muscle atrophy, lipid hydroperoxides and lipid mediators are considered key driving factors. The use of lipid hydroperoxide scavenger Liproxstatin-1 significantly alleviates muscle atrophy and mitochondrial oxidative damage following denervation (101). Complete denervation of the gastrocnemius muscle increases ROS formation, leading to increased lipid peroxidation, further protein degradation, and muscle atrophy. Substances such as neurobolil and thyroxin, which stimulate tissue growth and enhance antioxidant enzyme activity, have been shown to reduce muscle atrophy (102).

In specific disease models, bioactive compounds have demonstrated unique protective properties. In Duchenne muscular dystrophy (DMD) patients, He-Ne laser irradiation has been shown to alleviate oxidative stress by increasing nitric oxide (NO) levels and reducing lipid peroxidation, thereby slowing disease progression (103).

Szeto-Schiller peptides (SS peptides) are a class of engineered, mitochondria-targeted short peptides that bind to cardiolipin in the inner mitochondrial membrane, mitigating oxidative damage and improving mitochondrial function, thereby demonstrating potential protective effects in various disease models related to aging and tissue injury. SS peptides can interact with cell membranes to inhibit oxidative damage, showing potential in reducing oxidative stress and improving cellular health. Research indicates that the antioxidant properties of SS peptides make them valuable for the treatment of lipid peroxidation and sarcopenia (104, 105).

SS peptides, such as SS-31, may also enhance antioxidant defenses by modulating antioxidant enzyme systems like GPX4, helping to reduce lipid peroxidation and protect muscle cells from oxidative damage. Through this mechanism, SS peptides could improve muscle mass and delay the progression of muscle atrophy. While preliminary studies support the role of SS peptides in antioxidant and muscle protection, further clinical and animal research is needed to validate their therapeutic potential. Future studies should explore their mechanisms, especially in antioxidant, anti-inflammatory, and muscle metabolism regulation, to provide new treatment options for muscle atrophy induced by aging.

## 5 Conclusion and future directions

Lipid peroxidation plays a crucial role in sarcopenia, acting both as a key pathological driver and a potential therapeutic target. An increasing body of research suggests that lipid peroxidation products exacerbate degenerative muscle changes by inducing oxidative stress, disrupting cell membrane structures, impairing mitochondrial function, and regulating cell death pathways. However, interventions involving antioxidants and natural bioactive compounds—particularly those that effectively inhibit lipid peroxidation—have shown significant potential in slowing the progression of sarcopenia.

Future research should focus on elucidating the mechanisms by which lipid peroxidation affects different types of muscle fibers, as well as how gene regulation and metabolic interventions can precisely control lipid peroxidation levels. Combining lipid peroxidation-targeted therapies with other interventions, such as exercise training, nutritional supplementation, and electrical stimulation, could provide more effective solutions for the comprehensive management of sarcopenia. Moreover, developing new biomarkers to monitor the dynamic changes of lipid peroxidation in muscle health will be crucial for early diagnosis and intervention.

In summary, lipid peroxidation is both a promoter of sarcopenia pathology and a unique therapeutic entry point. A deeper understanding of lipid peroxidation mechanisms holds promise for providing more precise and effective treatment strategies, ultimately improving patients' quality of life and delaying muscle degeneration.
